# Mouse models of hepatocarcinogenesis: What can we learn for the prevention of human hepatocellular carcinoma?

**DOI:** 10.18632/oncotarget.170

**Published:** 2010-09-13

**Authors:** Mihael Vucur, Christoph Roderburg, Kira Bettermann, Frank Tacke, Mathias Heikenwalder, Christian Trautwein, Tom Luedde

**Affiliations:** ^1^Department of Internal Medicine III, University Hospital of RWTH Aachen, D- 52074 Aachen, Germany; ^2^Institute of Virology, Technical University München, Helmholtz Center München, D-81675 Munich, Germany

## Abstract

There is growing evidence that chronic inflammatory processes are involved in triggering the sequence from chronic liver injury to liver fibrosis, ultimately leading to liver cancer. In the last years this process has been recapitulated in a growing number of different mouse models. However, it has remained unclear whether and how these mouse models reflect the clinical reality of human hepatocellular carcinoma (HCC).

Research with animal models but also human liver specimens has indicated that the NF-&κB signaling pathway might withhold a crucial function in the mediation of chronic hepatic inflammation and the transition to HCC in humans. However, previous studies led to divergent and partly conflicting results with regards to the functional role of NF-&κB in hepatocarcinogenesis. Here, we discuss a new genetic mouse model for HCC, the liver-specific TAK1 knockout mouse, which lacks the NF-&κB activating kinase TAK1 specifically in parenchymal liver cells. Molecular findings in this mouse model and their possible significance for chemopreventive strategies against HCC are compared to other murine HCC models.

Hepatocellular carcinoma (HCC) represents the most common primary carcinoma of the liver [1]. In most instances, HCC arises in a setting of chronic inflammation and subsequent liver fibrosis [2]. Besides chronic alcohol consumption or drug abuse, autoimmunity or the uptake of liver toxins (e.g. Aflatoxin B1), infections with Hepatitis B- (HBV) and Hepatitis C-viruses (HCV) represent the main risk-factors for hepatocarcinogenesis [3, 4]. The world-wide spread of HBV and HCV not only in developing but also in industrialized countries has led to approximately 500 million people persistently infected with HBV or HCV. This resulted in a strong rise in HCC-incidence. Consequently, HCC is the 5^th^ most common cause for cancer related death world-wide; in some African or Asian countries HCC is even the first common cause for cancer related morbidity [3, 4]. In addition to its enormous clinical relevance, its unique pathophysiological features have made liver cancer research a field for studying basic molecular and cellular events driving chronic inflammation induced carcinogenesis, a process whose molecular underpinnings have largely remained elusive. Based on the wide range of these features, including immunology, tumor biology, genetics, metabolomics, cell biology etc., basic scientists from various research fields have focused their interest on the examination of animal HCC models. This will hopefully accelerate the discovery of new molecular mechanisms involved in hepatocarcinogenesis, subsequently leading to novel - urgently needed - therapeutic strategies against HCC.

Only recently, small inhibitor molecules have entered clinical practice to treat patients suffering from HCC. As such, Sorafenib (Nexavar®) is one of the new therapeutic agents that inhibit both pro-angiogenic (VEGFR-1, -2, -3; PDGFR-β) and tumorigenic (RET, Flt-3, c-Kit) receptor tyrosine kinases. Its efficacy in the context of HCC treatment was demonstrated in two large phase III clinical trials (SHARP and Asia-Pacific trial), which were conducted in both Western and Asian countries [5, 6]. Besides sorafenib, further therapeutic agents like regorafenib (BAY 73-4506) are currently investigated for their potential as anti-liver cancer therapeutics.

However, these new therapeutics “only” prolong survival and are palliative, while substances that could be used in an adjuvant setting are still lacking. In addition, it has to be clearly stated that HCC represent a diverse spectrum of cancers that will likely need - depending on the tumor type and tumor stage - different therapeutic strategies. In summary, systemic therapeutic options for HCC treatment are currently limited, underlining the need for new molecular targets. As outlined above, it has been well established that chronic inflammation and fibrosis precedes hepatocarcinogenesis. The eradication of the most common cause of chronic hepatic inflammation in humans (infection with HBV and HCV) is currently unattainable. Therefore, identification of central inflammatory signaling pathways that drive the transition from chronic liver injury to dysplasia and HCC might indeed open new possibilities for HCC-chemoprevention in a setting of chronic hepatitis.

In order to gain a better functional insight into the molecular mechanisms of hepatocarcinogenesis, multiple studies were performed using human HCC tissue. In the last years, a collection of genetic and epigenetic alterations, chromosomal aberrations, gene mutations and altered molecular pathways was described [7]. As such, chromosomal alterations could be attributed to certain genes potentially involved in hepatocarcinogenesis, such as c-Myc (8q), Cyclin A2 (4q), Cyclin D1 (11q), Rb1 (13q), AXIN1 (16p), p53 (17p), IGFR-II/M6PR (6q), p16 (9p), E-Cadherin (16q), SOCS (16p), and PTEN (10q) [7, 8]. Further, chromosomal alterations could be described in HCC, of which amplifications of 1q (58%-78%), 6p, 8q, 17q, and 20q, and deletions in 4q, 8p, 13q, 16q, and 17p represented the most frequent ones [9, 10]. However, in many cases it was difficult to assess whether these alterations represented a correlative epiphenomenon or if they were causally linked to HCC pathogenesis. In the light of the apsects mentioned above animal-models for HCC offer a unique possibility to study mechanistic and cellular aspects of tumor biology, including genetics of tumor initiation and promotion, tumor progression and spreading (metastasis) *in vivo*. Moreover, animal models also represent a valuable tool to pre-screen various therapeutic compounds for their efficacy to inhibit particular signaling pathways and prevent or decelerate HCC development.

The fact that inflammatory stimuli promote HCC development has been recapitulated in various rodent models and indeed during the last decades different models of chronic or acute liver damage induced carcinogenesis (e.g. chemically or genetically) have been established. One of the best studied chemically induced HCC-models is the diethylnitrosamine (DEN) induced liver carcinogenesis, which has been established in rats and mice [11]. In mice, a single dose of DEN at the age of 2 weeks causes DNA-damage, subsequent acute hepatitis, finally leading to HCC at approximately 8-10 months of age. The DEN rodent model withholds several advantages: (1) it can easily be administered to mice from different genetic backgrounds and of different genotype, (2) it has a high HCC incidence and (3) is highly reproducible [12]. Moreover, liver tumors from these mice withhold important features of a malignant cancer, since they metastasize in the lung. However, the primary event in DEN-induced carcinogenesis is DNA-damage leading to genetic mutations in an otherwise healthy liver. This indicates that - although happening in the context of acute liver damage - the sequence of primary DNA-damage and secondary acute inflammation does not quite reproduce the clinical reality of most patients, which develop HCC on the basis of chronic hepatitis in the absence of mutagens. Still, the DEN-rodent model has revealed crucial molecular and cellular pathways involved in the development of liver cancer and is a valuable tool to investigate particular molecules for their potential in inhibiting or promoting liver cancer formation [12-14].

In contrast to the DEN-rodent model, the Mdr2-knockout mouse represents a prototype of a genetically modified mouse model used to identify the pathways responsible for chronic inflammation-induced liver cancer. Mdr2-knockout mice lack a biliary transporter protein denoted as multi-drug resistance gene 2 (mdr2) leading to cholestatic hepatitis and liver cancer [15]. Tumor development in the Mdr2-knockout mice progresses through distinct phases: inflammation, dysplasia, dysplastic nodules, carcinoma and metastasis, thus mimicking to some degree the formation of HCC in humans [16]. A similar sequence of inflammation, mild fibrosis, dysplasia and HCC formation is observed in transgenic mice that overexpress the inflammatory cytokines lymphotoxin a and b (LTab) in the liver (AlbLTab) [17]. It was demonstrated that lymphotoxin LTa, b and their receptor (LTbR) are upregulated in livers of humans with HBV- or HCV-induced hepatitis and HCC. Subsequently, liver-specific LTab expression in mice induced liver inflammation and HCC [17]. In this context it is important to note that blockage of LTbR signaling strongly reduced the incidence of chronic hepatitis as well as abolished liver cancer [17].

The differences between the above mentioned rodent HCC-models are reflected by distinct, activated inflammatory signaling pathways leading to inflammation and hepatocarcinogenesis. One of the most important and best-studied inflammatory signaling cascades involved is - indeed - the NF-kB pathway. NF-kB can be activated by different stimuli like tumor necrosis factor or Interleukin-1 and controls the transcription of inflammatory and anti-apoptotic genes [18]. One important step in the activation of NF-kB is represented by activation of a high-molecular-weight kinase complex, the so-called IKK complex, consisting of two catalytic subunits, IKK1 (IKKa) and IKK2 (IKKb), as well as a regulatory subunit called NEMO (IKKg). Upon activation of this pathway by TNF, the IKK complex is recruited to adaptor proteins like TRAF2 and RIP1 and is activated by the kinase TAK1, so it can phosphorylate the inhibitory protein IkBa and mediate nuclear translocation of NF-kB (Fig. 1). In the DEN-rodent model, inhibition of NF-kB by conditional liver specific deletion of IKK2 led to increased liver-tumor formation [19], suggesting that the proinflammatory NF-kB signaling pathway suppresses hepatocarcinogenesis. In contrary, inhibition of the NF-kB signaling in the Mdr2-knockout mouse led to a strong decrease in hepatocarcinogenesis compared to mice with proficient NF-kB signaling, arguing for a tumor-promoter-function of this pathway [16]. Similarly, lack of NF-kB signaling strongly decreased chronic hepatitis and prevented hepatocarciniogenesis in the LT ab liver-tumor model, with the only difference that liver tumor formation also occurred in the absence of TNFR1 [17]. The reason for this obvious contradiction between the different murine HCC models has not been solved, meaning that it is presently not clear if NF-kB inhibition might be an option for chemoprevention of hepatocellular carcinoma in humans. Possibly, suppression of NF-kB signaling might be only beneficial for particular subtypes of human HCC – but this will need further investigation.

**Figure 1:**
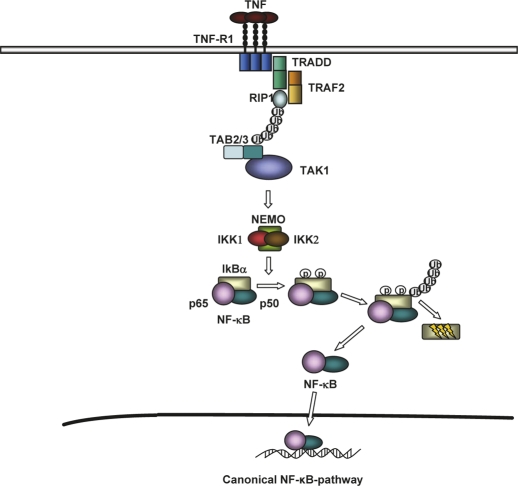
Activation of canonical NF-κB signaling by TNF-α Association of TNF-R1 results in TRADD-dependent TRAF2 and RIP1 recruitment. TRAF2 mediates K63-linked ubiquitination of RIP1 and recruits the IKK complex via the catalytic subunit NEMO. Autoubiquitination of TRAF2 causes TAK1 activation by interaction via TAB2/3. In cosequence TAK1 phosphorylate and activate IKKβ, which in turn phosphorylate IκBα, leading to his proteasomal degradation and releases NF-κB.

How can this contradictory role of IKKb signaling in HCC formation be reconciled? On the one hand IKKb signaling might be required for hepatocytes to respond to and survive carcinogenic stimuli and acute liver injury (e.g. DEN exposure). On the other hand, IKKb signaling might enable local chemokine expression by hepatocytes subsequently leading to chronic inflammation and HCC (e.g. like in the AlbLTab mouse model). Consistent with this hypothesis is the finding that AlbLTab x *Rag1*^-/-^ mice were devoid of chronic hepatitis, hepatocyte or oval-cell proliferation and failed to develop HCC.

Why could immune cells be essential for liver tumorigenesis? One explanation would be that CD4^+^ or CD8^+^ T-cells expressing inflammatory cytokines (e.g. IL1b, TNF, IFNg) as well as cytolytic proteins (e.g. Granzyme B) might contribute to hepatocyte cell death and tissue remodeling and transformation, finally leading to HCC [20]. Consequently, the role of NF-kB signaling in hepatocarcinogenesis might depend on the mouse model and the type or degree of liver inflammation and injury [21].

To further dissect the role of NF-kB in hepatocarcinogenesis we recently studied the roles of the molecules NEMO and TAK1 in hepatocarcinogenesis [22]. As mentioned above, both of these molecules are involved in controlling the activation of NF-kB in various cellular systems [22-24] and are important in mediating inflammatory signaling pathways. Surprisingly, conditional deletion of NEMO as well as TAK1 in hepatocytes (referred to as NEMO^LPC-KO^ and TAK1^LPC-KO^ mice) leads to spontaneous HCC-development in mice (Fig. 2A). Hepatocarcinogenesis was preceded by hepatic inflammation and liver cirrhosis which progresses through distinct phases (hepatitis, liver fibrosis and dysplasia) to HCC (Fig. 2B). These findings support the hypothesis that at least some members of the NF-kB pathway function as a tumor suppressors, and our further results suggested that this mainly confers to IKK subunits and molecules upstream of the IKK-complex. However, it is presently unclear if these new genetic tumor models mirror any entity of human HCC.

**Figure 2:**
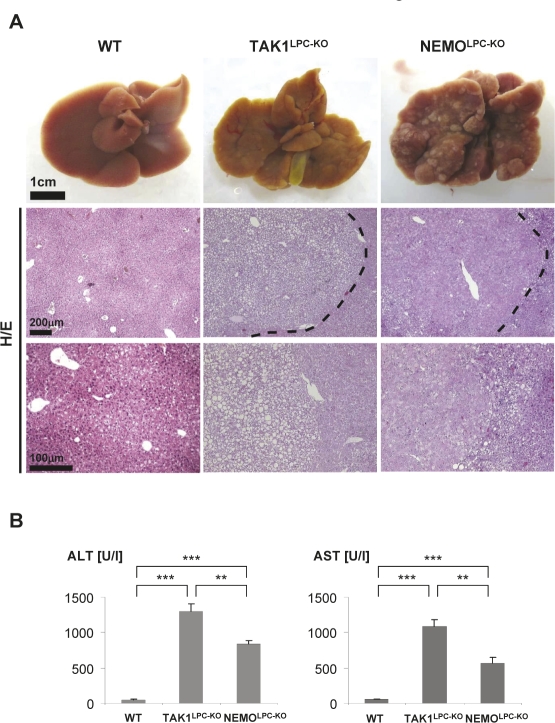
(A) Representative macroscopic pictures of 7-9 weeks-old male WT (left panel), TAK1^LPC-KO^ (middle panel) and NEMO^LPC-KO^ livers (right panel) Histological features of liver tumors in TAK1^LPC-KO^ and NEMOLPC-KO mice stained by H/E (lower panel, arrow head). TAK1^LPC-KO^ liver showed a clear cell HCC whereas NEMO^LPC-KO^ showed an eosinophilic, hepatoid HCC. (B) Serum level analysis of alanine aminotransferase (ALT), aspartate aminotransferase (AST). Results are shown as mean, error bars indicate SEM. **P<0.01, ***P<0.001 (n=5 each genotype).

HCC of TAK1^LPC-KO^ and NEMO^LPC-KO^ mice were investigated in further detail on genomic and transcriptional level. Therefore, we compared chromosomal aberrations in micro-dissected tumors of TAK1^LPC-KO^ and NEMO^LPC-KO^ mice. Comparative genomic hybridization analysis (aCGH) revealed that all liver tumors seen in NEMO^LPC-KO^ mice displayed chromosomal amplifications in a more or less random manner (data not shown). In contrast, TAK1^LPC-KO^ mice displayed defined chromosomal amplifications within certain hot-spot-areas on chromosomes 4, 8 and 13 [22]. These amplifications and deletions of chromosomal regions ranged from 0.68 megabase (MB) to 151 MB in TAK1^LPC-KO^ tumors and correlated with increased transcription of certain oncogenes located within the respective amplified chromosomal regions: Ntrk2, Net1 and Jun. These findings suggest completely different pathomechanisms of hepatocarcinogenesis between the TAK1- and NEMO-models: Although both mice show a defective NF-kB-activation in hepatocytes, only one model is associated with defined aberrations within certain chromosomal loci, upregulation of particular oncogenes as well as different histopathological features. This underlines that currently unknown molecular pathways downstream of TNF-associated molecules exist which might couple inflammation with specific genetic alterations as the basis for malignant transformation of hepatocytes. In this light, the new TAK1 model might represent an attractive model for a deeper understanding of these complex processes in the future, which might lead to a new chemopreventive strategy in patients with chronic hepatitis.

Given the rising number of genetic or chemical murine HCC models, the question remains if all these models are of significance for the understanding of human hepatocarcinogenesis and which of these models reflect which type of HCC in patients. This question could be addressed by a more systematic approach to compare specific molecular and genetic features between the different murine HCC models. As such, chromosomal aberrations in the various murine models, histopathology, transcriptional and metabolic changes could be compared at different stages of tumor initiation and promotion and correlated with the development of HCC in cirrhotic transformed livers. The same applies to signalome and transcriptome analyses or the possible role of hepatic stem cells during tumorigenesis.

The absence of a curative pharmacological treatment approach for human HCC and the limitations of the present molecular inhibitors in the palliative situation clearly underline the need for the identification of novel target molecules. The unique inflammation- carcinogenesis sequence in HCC suggest that such specific inhibitors against inflammatory signaling pathways may allow to intercept the continious transition from chronic liver injury to HCC. A systematic analysis of the various available murine liver cancer models might be the basis for the future identification of these potential targets and might open the door to the successful chemoprevention against liver cancer.
